# Childhood adversity, allostatic load, and adult mental health: Study protocol using the Avon Longitudinal Study of Parents and Children birth cohort

**DOI:** 10.3389/fpsyt.2022.976140

**Published:** 2023-01-05

**Authors:** Sabine Finlay, Robert-Paul Juster, Oyelola Adegboye, Donna Rudd, Brett McDermott, Zoltán Sarnyai

**Affiliations:** ^1^Laboratory of Psychiatric Neuroscience, Centre for Molecular Therapeutics, James Cook University, Townsville, QLD, Australia; ^2^Australian Institute of Tropical Health and Medicine, James Cook University, Townsville, QLD, Australia; ^3^College of Public Health, Medical & Veterinary Sciences, James Cook University, Townsville, QLD, Australia; ^4^Department of Psychiatry and Addiction, University of Montreal, Montreal, QC, Canada; ^5^Research Centre of the Montreal Mental Health University Institute, Montreal, QC, Canada; ^6^College of Medicine and Dentistry, James Cook University, Townsville, QLD, Australia

**Keywords:** mental health disorders, allostatic load (AL), ALSPAC, adverse childhood experiences, psychiatry

## Abstract

**Introduction:**

The cumulative burden of chronic stress and life events has been termed allostatic load. Elevated allostatic load indices are associated with different mental health conditions in adulthood. To date, however, the association between elevated allostatic load in childhood and later development of mental health conditions has not been investigated.

**Methods:**

Using data from the Avon Longitudinal Study of Parents and Children (ALSPAC), we will calculate allostatic load indices using biomarkers representing the cardiovascular, metabolic, immune, and neuroendocrine systems, at the ages of 9 and 17 years. Bivariate and multivariable logistic regression models will be used to investigate the association between allostatic load and psychiatric disorders in adulthood. Furthermore, the role of adverse childhood experiences as a modifier will be investigated.

**Discussion:**

This protocol describes a strategy for investigating the association between elevated allostatic load indices in childhood at the age of 9 years old and psychiatric disorders in adulthood at 24 years old.

## Background

The increased wear and tear on the body is the consequence of multisystem dysregulations in response to repeated challenges from stressful environment (1). This multisystem dysregulation can be objectively quantified by calculating a allostatic load index, a cumulative score of biological biomarkers representing the cardiovascular, immune, neuroendocrine, and metabolic systems (2, 3). Elevated allostatic load may be used to predict major health outcomes, both systemic and mental health (1, 2). Evidence suggests a link between an elevation of individual biomarkers in early childhood (9 years old) and the risk of mental health disorders in children, however, few immune biomarkers have been investigated (4).

We and others have shown that severe mental health disorders, such as schizophrenia, bipolar disorder, and psychotic disorders, are associated with increased allostatic load (3, 5–7). Furthermore, our group has established that elevated allostatic load is associated with depressive symptoms and may underlie higher mental health burdens and shortened life expectancy for populations affected by considerable stress and trauma, such as Indigenous Australian people (8, 9). Exposure to one adverse childhood experience has been shown to increase an individual's risk of early death through chronic disease, perhaps *via* a mechanism of impaired endocrine, neurological and immune system functions (10). A recent study conducted by our group, found an association between adverse childhood experiences, such as neglect, physical and sexual abuse, and elevated allostatic load in adulthood (11). Because of these findings, we will investigate adverse childhood experiences as a modifier for the effect of allostatic load in childhood and the development of psychiatric disorders at the ages of 17–24 years. We hypothesize that the neurobiological impact of the adverse childhood experience may influence the developmental trajectories of both the stress-related physiological systems, resulting in elevated allostatic load, and the brain, to give rise to psychiatric disorders in adulthood.

This project will expand upon our earlier findings and include biological markers from the four major physiological systems to calculate the allostatic load index and to investigate an association between multisystem dysregulation in childhood and the development of severe mental health disorders in adulthood. If such an association is found, then allostatic load indices could be applied for targeting interventions in young populations at risk and aiming to reduce the risk of developing a mental health disorders in adulthood.

## Aims

This protocol describes a strategy for investigating the association between elevated allostatic load indices in childhood (age 9 years old and 17 years) and psychiatric disorders in adulthood (age 24 years old). This investigation will use longitudinal data from the Avon Longitudinal Study on Parents and Children (ALSPC), as this data set allows us to calculate allostatic load indices based on selected biomarkers in the cardiovascular, immune, neuroendocrine, and metabolic systems. Furthermore, the project will investigate the modifying role of adverse childhood experiences on allostatic load at ages 9 and 17 years and the later development of mental health disorders.

## Objectives

1) Investigate and model the association between allostatic load at age 9 years and the development of psychotic disorders (schizophrenia, psychosis, and bipolar disorder) or psychotic symptoms at age 24 years.
2) Investigate and model the association between allostatic load at age 9 years and the development of mood disorders (depression and anxiety) at age 24 years.
3) Investigate and model the association between allostatic load at age 17 years and the development of psychiatric (both psychotic and mood) disorders in adulthood.
4) Investigate whether adverse childhood experiences during childhood modify the relationship between allostatic load at age 9 and 17 years and the development of psychiatric (both psychotic and mood) disorders in adulthood.
5) Investigate the role of adverse childhood experiences (sexual abuse, physical abuse and neglect) in developing psychotic and mood disorders in adulthood.

## Methods

### Source of data (ALSPAC)

This study will use a large cohort data from the Avon Longitudinal Study of Parents and Children (ALSPAC) (12). The ALSPAC is a prospective observational study that recruited pregnant women in and around the City of Bristol, United Kingdom, with expected delivery dates from the 1^st^ of April 1991 to the 31^st^ of December 1992 (13). Phase I recruitment (initial) included 14,541 pregnancies (14,676 fetuses, resulting in 14,062 live births and 13,988 children who were alive at 1 year old). By the time the children from Phase I were approximately seven years, the study was expanded by recruiting eligible cases who had not joined the initial study. By the time children from Phase I were 24 years old, an additional 913 index children had been recruited through Phase II (456 children), Phase III (262 children), and Phase IV (195 children). Therefore, the total recruited sample available for analysis is 15,454 pregnancies or 15,589 fetuses; of these, 14,901 were alive at 1 year old. Frequent assessments of participants have been conducted with 68 data collection points between birth and 18 years of age (14). In Phase I, 50.3% of the children were male and 96.1% were of White ethnicity.

Study data were collected and managed using REDCap electronic data capture tools hosted at the University of Bristol. REDCap (Research Electronic Data Capture) is a secure, web-based software platform designed to support data capture for research studies (15). We will derive a clinical model using participant-reported data from the ALSPAC.

### Participants

Study participants come from the ALSPAC dataset using pre-specified eligibility criteria. For inclusion, the participants will:

- Have available biological data to accurately calculate allostatic load at 9 and/or 17 years.
- Alive at 1 year of age.
- Have information available regarding their mental health at age 17–24 years.
- Have available information about childhood neglect, childhood sexual abuse, or childhood physical abuse.

**Figure 1:**
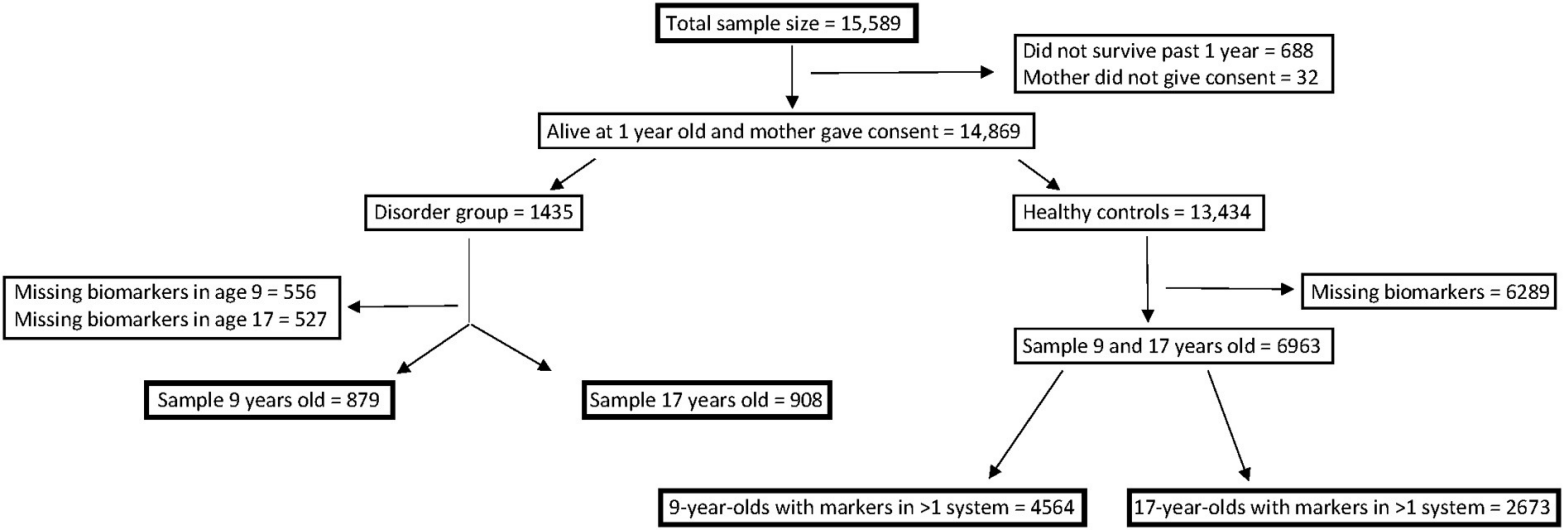
Process of how sample groups were divided, including exclusion numbers.

Figure 1 shows the expected selection process. Many participants in the ALSPAC study were missing biomarkers in all but one system (6,289 individuals in the “healthy control” group and 1,083 in the “disorder group”) and were therefore excluded.

### Primary outcome variable of interest

The primary outcome of interest is the presence of a mental health disorder diagnosed in adulthood (22–24 years). Whenever outcome is measured at the age of 17, such as met diagnosis for psychotic disorder and anxiety, exposure will be considered at age 9 only.

Psychotic experiences (PE) were identified at the age of 24 years through the face-to-face, semi-structured Psychosis-Like Symptom Interview (PLIKSi) conducted by psychology graduates in assessment clinics. Following this, they were coded according to the definitions and rating rules for the Schedules for Clinical Assessment in Neuropsychiatry (0 = none, 1 = psychotic symptoms). At age 17 years, the individuals were assessed for “met diagnosis for psychotic disorder” with a dichotomous answer (yes/no).

Depression was assessed at the age of 22 years using the short version of the “Mood and Feelings Questionnaire” (MFQ), which includes 13 questions. The MFQ scale is 0–26, as questions can be given the scores 0 = “not true,” 1 = “somewhat true” and 2 = “true.” Scores > 12 are suggestive of likely depression. Also, at 22 years, participants were asked [with a dichotomous answer (yes/no)] if they had ever been diagnosed with: schizophrenia; bipolar disorder; or depression. Anxiety was assessed at the age of 17 years using The Clinical Interview Schedule-Revised (CIS-R). The CIS-R is a structured interview for the measurement and diagnosis of non-psychotic, psychiatric morbidity developed by Lewis et al. (16). The scale used by ALSPAC was from 0 to 4; 0 = not present, 1 = mild, 2 = moderate, 3 = severe, and 4 = very severe.

### Primary exposure

The primary exposure of interest is the allostatic load index at the age of 9 and 17 years old, while adverse childhood experience will be used as a modifier.

### Description of primary exposure variables

#### Anthropometric biomarkers

From age 7 years, all children were invited to attend clinics yearly to assess height and weight. Bodyweight was measured using electronic scales, and standing height was measured by a stadiometer (Leicester height measure, Child Growth Foundation, London, UK). The body mass index (BMI) was calculated using these results.

#### Cardiovascular biomarkers

At each visit to the clinic, blood pressure was measured using a Dinamap 9301 Vital Signs Monitor (Morton Medical, London, UK). Blood pressure was measured from the right arm while the participant was seated and at rest. An appropriate cuff size was used, and the participant's arm was supported during measurement. Two readings were collected for systolic and diastolic blood pressure. The mean of the two measurements was used in the ALSPAC database. Heart rate at the age of 9 years was assessed during a predicted work capacity at a heart rate of 170 bpm. Heart rate at the age of 17 years was assessed at a resting position.

#### Metabolic biomarkers

Non-fasting peripheral blood was collected in children at the age of 9 years old. Samples were spun within 4 h and stored at −80°C until analysis. The samples were assayed in 2008 after a median of 7.5 years in storage with no previous thaw during this period. Metabolomic profiling was performed using the Nightingale NMR metabolomics platform (Helsinki, Finland). From the non-fasting samples, triglycerides, high-density lipoprotein (HDL-c), low-density lipoprotein (LDL-c), total cholesterol (TC), insulin, and very-low-density lipoprotein (vLDL) were measured. Serum insulin was measured with an enzyme-linked immunosorbent assay (ELISA, Mercodia, Uppsala, Sweden), that does not cross-react with proinsulin. Plasma glucose was measured with an automated assay. Plasma triglycerides, total cholesterol, and HDL concentration were measured by a modification of the standard Lipid Research Clinics Protocol by using enzymatic reagents for lipid determination and LDL concentration was determined from these, using the Friedwald equation (17). Inter and intra-assay coefficients of variation for insulin were <9.3 and <6.0%, respectively and for lipids and glucose, both inter-and intra-assays were all <5%.

Fasting peripheral blood was collected at ages 17 and 9 years (sub-study BSS). From the fasting samples, insulin, glucose, triglycerides, HDL-c, LDL-c, and TC were collected. Fasting triglycerides, cholesterol, and HDL levels were measured on a Dimension RXL system (Dade Behring) using reagents supplied by the manufacturer. Inter- and intra-assay coefficients of variations were <4 %. Insulin was measured by ELISA using a commercial kit (DSL, London, UK). Sensitivity was 0.26 mU/l. Intra-assay CVs were 4.4% and 5.1% at 10.3 and 35.8 mU/l, and equivalent inter-assay coefficients of variations were 8.7% and 2.9%; this assay has no cross-reactivity with proinsulin at levels up to 1,000 pmol/l. Glucose was measured by the glucose oxidase method on a YSI 2300 stat plus analyser (YSI, Farnborough, Hants, UK). The intra-assay coefficients of variations were 1.5% at 4.1 mmol/l, and the inter-assay coefficients of variations were 2.8 and 1.7% at 4.1 and 14.1 mmol/l respectively. Fasting vLDL was available at the age of 17 years. Glycosylated hemoglobin (HbA1c) reflects the average blood glucose over the lifespan of the red blood cells (approximately 3 months) and was measured at the age of 9 years by ion-exchange HPLC assay using the HA-8140 Hi-Auto HbA1c analyser (Menarini Diagnostics), maintained in alignment with the Diabetes Control and Complications Trial (DCCT) method. Inter-assay coefficients of variations were 1.8% for a mean HbA1c of 5.5 and 1.9% for a mean HbA1c of 9.7% throughout the study. Adiponectin was measured by enzyme-linked immunosorbent assay (R&D Systems, Abingdon, UK) at ages 9 and 15 years.

#### Immune biomarkers

Interleukin-6 (IL-6) and C-reactive protein (CRP) levels were assayed using ELISA (R&D Systems, Abingdon, UK) and automated particle-enhanced immunoturbidimetric assay (Roche UK, Welwyn Garden City, UK), respectively. All inter-assay and intra-assay coefficients of variation for IL-6 and CRP were <5%. IL-6 was measured at time point 9 years old, whereas CRP levels were measured at both 9 and 17 years of age. Albumin concentration was measured at age 9 years using a standard laboratory method on a Roche Modular analyser (Roche Diagnostics Ltd, West Sussex, UK).

#### Neuroendocrine biomarkers

Cortisol and dehydroepiandrosterone sulfate (DHEAS) were measured at age 9 years through the BBS sub-study. The BBS was a sub-study of the Children in Focus (CIF) cohort in which, fasting blood and urine were collected. The CIF cohort was chosen at random from the last 6 months of ALSPAC births, occurring from the 6^th^ of June to the 11^th^ of December 1992. DHEAS was assayed automatically by immunochemiluminescence (Immulite assay, DPC, Madrid, Spain). Intra- and inter-assay CVs were 5.6 and 10.1%, respectively. Cortisol was measured by immunochemiluminescence (Cortisol ELISA assay, DSL). Intra- and inter-assay CVs were 2.4 and 6.1%, respectively.

### Adverse childhood experience

One of the main variables of interest was an aggregated score assessing adverse childhood experiences assessed at age 11 when participants (the children) were allowed to answer for themselves. Adverse childhood experience was measured *via* childhood neglect, childhood physical abuse and childhood sexual abuse. Childhood neglect was assessed with the following questions: (1) *Frequency adults in the family shouted at the respondent before age of 11* and (2) *frequency adults in the family said hurtful or insulting things to the respondent before age of 11*. These questions were based on a scale 1 to 5; 1 = never, 2 = rarely, 3 = sometimes, 4 = often, and 5 = very often. Childhood physical abuse was assessed with the questions: (1) *Frequency adults in the family actually kicked, punched, or hit respondent with something that could hurt respondent or physically attacked respondent in another way before age of 11*, and (2) *frequency of adults in the family hit respondent so hard it left bruises or marks before the age of 11*. The same scale as the childhood neglect category was used. Childhood sexual abuse was assessed with the questions: (1) *Respondent was touched in a sexual way by an adult or older child or was forced to touch adult or older child in a sexual way, before age of 11* and (2) *adult or older child forced or attempted to force, respondent into any sexual activity by threatening or holding respondent down or hurting respondent in some way, before the age of 11, rated on the scale*1 to 3; 1 = no, this did not happen before 11, 2 = yes, this happened once, 3 = yes, this happened more than once.

**Figure 2:**
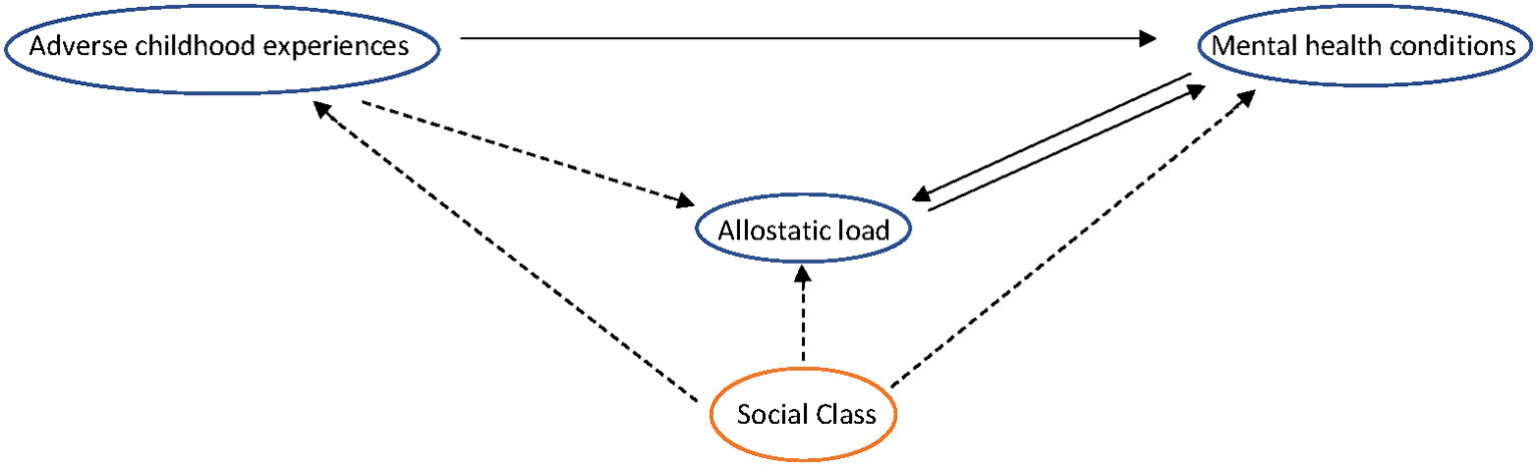
A conceptual model of the association between adverse childhood experiences, allostatic load, and mental health conditions.

A conceptual model was designed to show the possible association between adverse childhood experiences, allostatic load, and mental health conditions. Social class was depicted as a possible confounder, as it can impact all three factors (Figure 2).

### Other variables

Other variables of interest used as cofounders were (1) sex of the participants (male/female), (2) mother's occupation, (3) age of mother when giving birth, and (4) gestational age at birth. Sex was recorded at birth and treated as a binary variable. The mother's occupation was recorded at birth using the UK Office of National Statistics' socioeconomic classification system (Class I = professional; Class II = managerial and technical workers; IIIa = skilled non-manual occupations; IIIb = skilled manual occupations; IV = partly skilled occupations; V = unskilled occupations).

#### Data management and data analysis

Statistical analysis will be conducted in IBM SPSS version 25, Microsoft Excel, and *R* version 4.0.2.

The data will constitute carefully selected biomarkers, which include cardiovascular, immune, neuroendocrine, and metabolic biomarkers that can be used to calculate the allostatic load index in participants at age 8.5–9 years (will be referred to as 9 years of age from now on) and age 17 years. The biomarkers are selected based on previous research. Furthermore, we will acquire data on the diagnosis of mood disorders at the age of 17–22 years old and psychotic disorders at the age of 22–24 years old. We will create the allostatic load index for each participant at age 9 years, and this will be used to investigate the likelihood of an individual developing a psychiatric disorder at age 17–24, which includes schizophrenia, psychosis, bipolar disorder, anxiety, and/or depression, respectively.

#### Calculation of the allostatic load

The allostatic load index will be calculated using two approaches common in the literature (18). The first method is the traditional count-based “Group allostatic load index” with summary measures representing the number of biomarkers falling within a high-risk percentile (i.e., exceeding the 75^th^ percentile or below the 25^th^ percentile) based on the healthy controls' distribution of biomarker value. Each biomarker can have either the value 0 (normal range) or 1 (abnormal range), resulting in each biomarker being allotted an equal weight in the index. This is the standardized approach in the allostatic load literature (19). The second method is the “z-score allostatic load index” with summary measures representing the sum of an individual's obtained z-scores for each biomarker based on the distribution of values in the healthy controls. This method allows the weight of each biomarker to be different depending on its deviation from the healthy controls' mean (18). Using these approaches, participants' allostatic load can be grouped into tertiles based on the allostatic load index threshold, this approach is called the “Nominal allostatic load grouping.” Thus, individuals with allostatic load index scores in the top and middle thirds can be compared to those in the bottom thirds (18). This method converts the discrete groups for comparison.

Therefore in this study, allostatic load in participants aged 9 and 17 years will be calculated based on available parameters representing the anthropometric, cardiovascular, metabolic, immune, and neuroendocrine systems. The biomarkers were selected based on previous research for monitoring physiological dysregulations (3, 20, 21). All three methods described will be applied to our data. Collectively and respectively, our formulations allow for ordinal, continuous, and categorical operational definitions of allostatic load.

#### Missing data

Different approaches to dealing with missing data will be considered. Participants with biomarkers in only one biological system will be excluded. To account for further missing data, we will use multiple imputations by chained equations (MICE). MICE will be implemented in *R* and is a flexible and practical approach to handling missing data. MICE produce asymptotically unbiased estimates and standard errors and is asymptotically efficient when correctly implemented (22). We will carry out a sensitivity analysis after the imputation of missing data.

#### Data analysis

Preliminary descriptive analyses will include frequencies and percentages for categorical variables, mean, standard deviation, median and interquartile range for continuous variables. Generalized linear models will be used to fit models to the ALSPAC data. For example, bivariate and multivariable logistic regression will be used to model whether a person develops a mood (and psychotic) disorder or not separately. Multiple linear regression will be applied to continuous variables such as the mood disorder index. All models will be adjusted for confounders. Additional analysis will be carried out to investigate the effects of modifying variables on the outcome variable (e.g., mood and psychotic disorder). For binary outcomes, marginal effects in addition to odds ratios (ORs) and adjusted odds ratio (aOR) will be reported. Marginal effects will allow the interpretation of the original probability scale, which is more interpretable. The effect modification was investigated by including the interaction term between allostatic load and childhood experience. A modification is established if a statistically significant association between allostatic load and outcome (e.g., psychiatric disorder) differs with levels of childhood experience in the regression analysis.

We will consider several models in this study. The first multiple linear regression will investigate the effect of allostatic load and sex on the psychiatric outcome, either as a psychotic disorder using binary outcomes or a mood disorder using a combination of binary (“have you ever been diagnosed with depression, schizophrenia or psychosis”– yes/no), ordinal (anxiety scale) and continuous (MFQ scale) outcomes. In the second model, the effect of allostatic load and social class on these psychiatric symptoms (both psychotic and mood) will be explored, while the last model will include psychiatric outcomes (using a combination of binary, ordinal and continuous outcomes), allostatic load, sex, and social class.

Participants in the “disorder group” (age 9 and 17 years) will be divided into tertiles according to the distribution of allostatic load. We will use logistic regression to examine psychiatric outcomes in adolescence/early adulthood among individuals in the middle and top thirds compared with the bottom third of allostatic load distribution. Furthermore, a comparison of individuals in the top and bottom tertiles will be calculated. Linearity of the association will be determined by inspection of the ORs over the thirds of the allostatic load distribution. Linear mixed models will also be fitted to the data to adjust for repeated measurements at ages 9 and 17.

## Discussion

This protocol has been developed based on the findings of our systematic review, which was performed to investigate the relationship between allostatic load and the risk for children and young adults of developing psychiatric disorders in later life. We intend to include a broad range of biological markers to accurately assess allostatic load at different timepoints (age 9 and 17 years) in individuals that later did (patients) or did not (healthy controls) develop either a mood or psychotic disorder. Important variables, such as adverse childhood experiences, which cover neglect, and physical and sexual abuse will also be investigated as a possible modifier of the association between allostatic load and the development of psychiatric disorders. This research has the potential for great impact, as it may help to identifyindividuals in childhood/early adulthood that are at increased risk of developing psychotic and mood disorders in later life, allowing for early intervention and recognition.

Currently, there is no consensus regarding the calculation of the allostatic load index (23). This study calculated the allostatic load index using different biomarkers from the four biological systems (cardiovascular, metabolic, neuroendocrine, and immune). Twenty-three biomarkers (three cardiovascular, 11 metabolic, two neuroendocrine, and two immune biomarkers) at the age of 9 years and 13 biomarkers (three cardiovascular, nine metabolic, and one immune biomarker) at the age of 17 (age 15 years for Adiponectin) were selected. This protocol builds on the findings from our systematic review to use and validate the allostatic load index as a determinant for the increased risk of developing psychiatric disorders in early adulthood. We intend to include a broad range of biomarkers and psychiatric disorders, along with the sex, and occupational status of mothers (for socioeconomic status), and different adverse childhood experiences, such as neglect, sexual and physical abuse. We have a plan to deal with missing data, assess models, and use various statistical analytic models.

As an additional analysis, the magnitude of effect from the allostatic load index will be compared to the common single biomarkers, CRP, and IL-6. We will assess if the allostatic load index provides a better goodness-of-fit than the use of just one biomarker.

Because ALSPAC data was based on extracted coded information from the medical records due to concerns that free-text notes may pose a confidentiality risk, our analysis will only use coded data and thus cannot take advantage of the increase in algorithm sensitivity that using free text brings.

Limitations of the study include missing data, a well-known challenge for prospective cohort studies. When possible, the missing data will be handled by using sensitivity analysis, including multiple imputations for missing data. Another method to handle missing data is excluding all participants that do not have a full set of data (biomarkers and information about mental health disorders). The neuroendocrine (stress) biomarkers, cortisol and DHEAS, were only measured in a subsample (BBS) of children at the age of 9 years. Including these two important biomarkers will result in a significant decrease in our sample size. Another limitation is the time point of diagnosis of mental health disorders, especially bipolar disorder and schizophrenia. For the ALSPAC sample, this was diagnosed at the age of 22 years old, however, the diagnoses most commonly occur in the twenties for bipolar disorder (24) and mid-to late 20's for schizophrenia (25). As a result, individuals who have not developed symptoms at 22 years old will be considered healthy controls, underestimating the potential link between allostatic load and psychotic symptoms.

A potential risk or disadvantage for prospective cohort studies is their challenges in detecting “rare” diseases. The psychotic mental health disorders, schizophrenia, bipolar disorder and psychosis are only prevalent in 0.4% (26), 0.6–0.9% (27) and 0.39% (28) of the general population, respectively. This may result in very small case numbers for this study.

In conclusion, this protocol describes for the first time a strategy to investigate the potential association between elevated allostatic load, a cumulative index of multisystem dysregulation in response to challenges to the organism, in childhood and psychiatric disorders and symptoms in young adults.

## Ethics statement

The studies involving human participants were reviewed and approved by ALSPAC Ethics and Law Committee and the Local Research Ethics Committees, Human Research Ethics Committee at James Cook University, Australia. Written informed consent to participate in this study was provided by the participants' legal guardian/next of kin.

## Author contributions

SF and ZS conceived the overall concept of the study and were the main contributors in writing the manuscript. R-PJ, OA, DR, and BM were involved in the study design. SF is the grant holder. R-PJ provided expertise in the allostatic load and insight into different methods to calculate this. OA provided statistical expertise in analyzing the data. DR provided expertise in biomarkers and their biological significance. All authors contributed to the refinement of the study protocol and approved the final manuscript.
